# Mechanochemistry in Portugal—A Step towards Sustainable Chemical Synthesis

**DOI:** 10.3390/molecules27010241

**Published:** 2021-12-31

**Authors:** Vânia André, M. Teresa Duarte, Clara S. B. Gomes, Mafalda C. Sarraguça

**Affiliations:** 1 Centro de Química Estrutural, Instituto Superior Técnico, Universidade de Lisboa, Av. Rovisco Pais 1, 1049-001 Lisbon, Portugal; 2 Associação do Instituto Superior Técnico para a Investigação e Desenvolvimento (IST-ID), Av. Rovisco Pais 1, 1049-003 Lisbon, Portugal; 3 LAQV-REQUIMTE, Departamento de Química, NOVA School of Science and Technology, 2829-516 Caparica, Portugal; 4 Associate Laboratory i4HB—Institute for Health and Bioeconomy, School of Science and Technology, NOVA University Lisbon, 2819-516 Caparica, Portugal; 5 UCIBIO—Applied Molecular Biosciences Unit, Department of Chemistry, School of Science and Technology, NOVA University Lisbon, 2819-516 Caparica, Portugal; 6 LAQV-REQUIMTE, Departamento de Ciências Químicas, Faculdade de Farmácia, Universidade do Porto, Rua Jorge Viterbo Ferreira, 228, 4050-313 Porto, Portugal

**Keywords:** mechanochemistry, ball milling, supramolecular synthesis, green chemistry, history, Portugal

## Abstract

In Portugal, publications with mechanochemical methods date back to 2009, with the report on mechanochemical strategies for the synthesis of metallopharmaceuticals. Since then, mechanochemical applications have grown in Portugal, spanning several fields, mainly crystal engineering and supramolecular chemistry, catalysis, and organic and inorganic chemistry. The area with the most increased development is the synthesis of multicomponent crystal forms, with several groups synthesizing solvates, salts, and cocrystals in which the main objective was to improve physical properties of the active pharmaceutical ingredients. Recently, non-crystalline materials, such as ionic liquids and amorphous solid dispersions, have also been studied using mechanochemical methods. An area that is in expansion is the use of mechanochemical synthesis of bioinspired metal-organic frameworks with an emphasis in antibiotic coordination frameworks. The use of mechanochemistry for catalysis and organic and inorganic synthesis has also grown due to the synthetic advantages, ease of synthesis, scalability, sustainability, and, in the majority of cases, the superior properties of the synthesized materials. It can be easily concluded that mechanochemistry is expanding in Portugal in diverse research areas.

## 1. Introduction

Green chemistry is based on the set of principles defined by Paul Anastas in 1988 [1]. These twelve principles promote the reduction or elimination of use or generation of hazardous substances in the design, manufacture, and application of chemical products. Increasing concern about climate change and the establishment of the 17 Sustainable Development Goals by the United Nations demand an urgent paradigm shift both in scientific research and the industrial sector towards more sustainable and more environmentally friendly technologies [2]. Nowadays, the world is facing numerous societal and economical challenges, from no net emissions of greenhouse gases to economic growth decoupled from resources usage. To face these challenges, it is crucial to reduce waste and create a more sustainable and “greener” industry. A growing interest in the design of low-environmental-impact processes has led to the search for alternative and innovative technologies capable of complying with recent guidelines, such as the European Green Deal [3]. The European Green Deal provides an action plan to enhance the efficient use of resources by shifting to a clean, circular economy to restore biodiversity and reduce pollution. Two of the points that require action are investment in environmentally friendly technologies and support of industry innovation. Industry and academia have become more conscious of the importance of adopting more sustainable and environmentally friendly chemical processes, using safer solvents, and preventing waste [4]. 

In recent years, mechanochemistry [5] has grown as a promising alternative synthetic method in the various areas of inorganic, organic, and metal-organic chemistry. Mechanochemistry has shown to be capable of activating chemical transformations by mechanical forces, e.g., compression, shear, or friction in the solid phase, without the need of solvents, or for the use of trace amounts of solvents [6,7,8].

Solid-state reactions allow for the exploration of new synthetic pathways, which can lead to better control over stereoselectivity, stoichiometric efficiency, atom economy, and formation of nanotechnological products. Moreover, different reaction processes have led to the investigation of and access to poorly soluble but cheaper reactants or new raw materials. Strong covalent bonds, such as carbon-carbon and carbon-heteroatom (C-N, C-O, C-halogen, C-S, C-Se, and C-Te), can be activated by solid-state grinding under mild conditions of temperature and reagents. Examples of organic covalent reactions performed by mechanochemistry include dehydrogenative coupling, oxidation, reduction, and organocatalytic reactions [9,10,11,12,13,14]. Mechanochemistry has also been showing promising advances in organometallic synthesis [15].

Crystal engineering and supramolecular chemistry have also largely benefited from the use of mechanochemistry, as proven by the increasing number of publications reporting the synthesis of cocrystals, salts, metal-organic frameworks (MOFs), covalent-organic frameworks (COFs), and coordination polymers (CPs) by mechanochemical methods [16,17,18,19,20]. The relevance of this technique is even more evident in the formation of pharmaceutical cocrystals, which can usually be better achieved by solid-state reactions rather than (re)crystallization from solution, as the milling processes are known to help to overcome the problem of the poor solubility of active pharmaceutical ingredients (API) [21,22].

Due to its characteristics, mechanochemistry is an emerging technique with potential to change the current dominance of wet chemical synthesis that is associated with sustainability; it was listed by IUPAC as one of the top 10 chemical innovations towards a sustainable future [23,24,25].

In 2019, a COST Action focused on “mechanochemistry for sustainable industry” (CA18112, MechSustInd) [26] was initiated, aiming to promote fundamental and applied research in mechanochemistry and its implementation in European industry. This collaborative network is composed of partners from 33 COST-member countries, including Portugal and the team involved in this review, as well as 17 other Inclusiveness Target Countries and partners worldwide. Currently, MechSustInd is represented by a total of 77 academic and other institutions in Europe and around the world.

### 1.1. Mechanochemical Methods

Mechanochemical synthesis may be conducted under different conditions that often influence the outcome and the success of the reaction. The use of mechanochemical ball milling for the synthesis of the different types of compounds has been extensively reported over the last years. Amongst the different available mills, the most common are vibrational and planetary mills, with the latter being prone to scale-up, while the former is more suitable for small-scale synthesis. While in vibrational mills, jars swipe back and forth at a given frequency, in planetary ball mills, the jars rotate around a central axis and, at the same time, they spin around their own axis, creating centrifugal forces.

**Figure 1:**
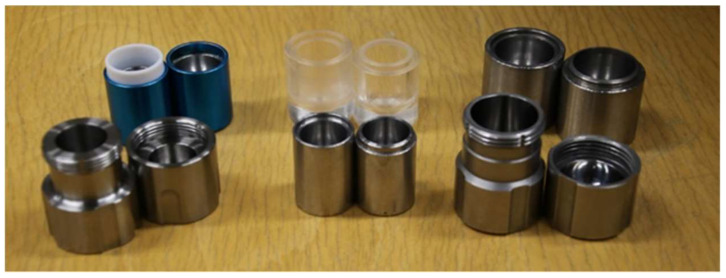
Examples of screw and snap-closure stainless steel and PMMA grinding jars for vibrational mills.

Stainless steel is the most common material used for the grinding jars and balls, but this may introduce metal contamination in the final bulk product. Available alternative materials are zirconia, tungsten carbide, polytetrafluoroethylene (Teflon), and poly(methyl)methacrylate (PMMA) (Figure 1).

From the point of view of designing the best strategy, it is also important to retain that the addition of catalytic substances (e.g., solvent) may control the reactivity. Besides grinding without the addition of any other compound other than the reagents (neat grinding—NG), the use of catalytic amounts of a solvent (liquid-assisted grinding—LAG) is quite often reported to accelerate or even enable reactions that would not occur by neat grinding. The definition of LAG is based on how mechanochemical reactivity is affected by the ratio of the liquid additive to the weight of reactants (η). An η value of 0 corresponds to neat grinding; if η > 10 μL/mg, then we have a solution reaction. In LAG, the η value lies in the range of ≈0–1 μL/mg. In this range, reactivity appears independent of reactant solubility, distinguishing LAG from slurry reactions (η > 1 μL/mg), in which low solubility can hinder reactivity [27]. The addition of catalytic amounts of ionic salts (ion- and liquid-assisted grinding—ILAG) [28,29] and polymer (polymer-assisted grinding—POLAG) [30,31] is also known to play a role in some mechanochemical reactions.

Regardless of the success obtained with these techniques at lab scale, the scalability of such processes poses an issue for industrial applications. Even though the mechanochemical synthesis of drug-carrier composites at the 50 kg scale has been reported to be successful by ball milling, the typical values range from tens to hundreds of grams, and thus, mechanochemical synthesis by ball milling at large scale has not yet been fully proven [32]. Twin screw extrusion (TSE) has been presented as an alternative mechanochemical approach for the continuous production of different compounds (such as cocrystals and MOFs) with high yield, purity, and crystallinity at kg·h^−1^ rates with no added solvent or only minimal added solvent [32].

### 1.2. Mechanochemistry in Portugal

The first publications regarding mechanochemistry being developed by Portuguese researchers date back to 2009 and 2010 and resulted from collaborations between the groups of Duarte, Braga, and Friščić at the Universities of Bologna and Cambridge, respectively. These first studies were based on the development of mechanochemical strategies for the synthesis of metallopharmaceuticals [33] and metallodrugs [34]. The ILAG synthesis of bismuth subsalicylate was an important step in the field, as it can be considered the first report on the success of this type of synthesis of a commercially available metallodrug, leading to shorter reaction times, milder conditions, higher yields, ease of the process, and a much more environment friendly process [34].

**Figure 2:**
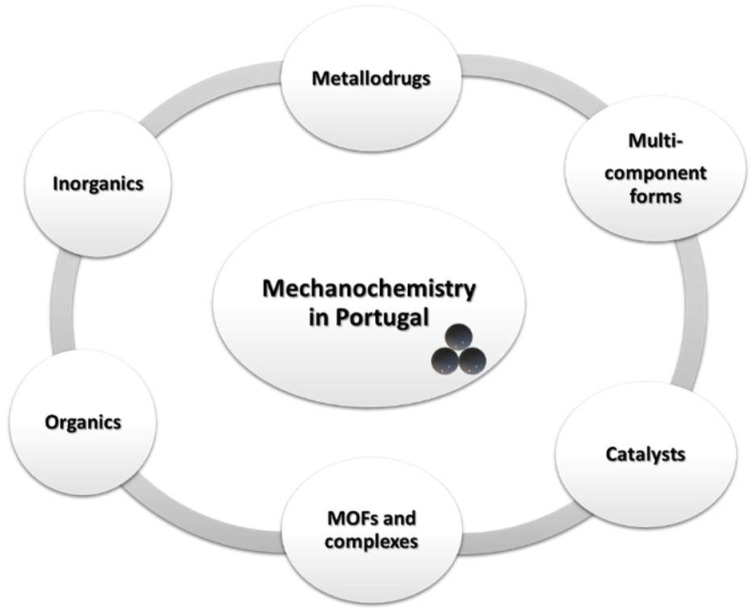
Applications of mechanochemistry in Portugal.

Almost simultaneously, the application of mechanochemistry to the synthesis of multicomponent crystal forms also sparked interest among Portuguese researchers (Figure 2), especially regarding active pharmaceutical ingredients. This has been an area of growing interest in Portugal, with several research groups devoting their efforts towards the design and synthesis of solvates, salts, and cocrystals. More recently, the synthesis of pharmaceutical ionic liquids via these methods has also been reported as possible [35].

Another main branch of mechanochemistry studies in Portugal involves the formation of complexes and metal-organic frameworks (MOFs) [36]. Application towards catalysts is also currently being explored [37].

## 2. Developments of Mechanochemistry in Portugal

### 2.1. Mechanochemistry in the Synthesis of Polymorphs and Multicomponent Forms

**Figure 3:**
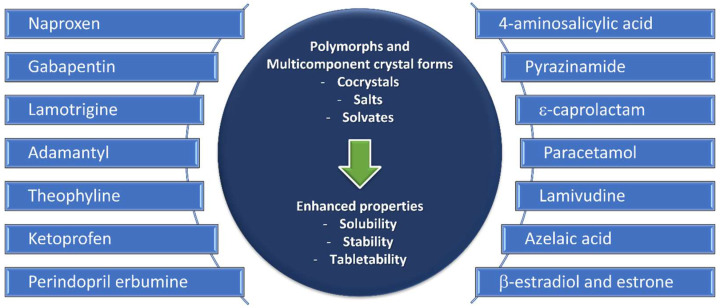
Polymorphs and multicomponent forms discussed in this review.

Crystal Engineering comprises “the understanding of intermolecular interactions in the context of crystal packing and the utilization of such understanding in the design of new solids with desired physical and chemical properties”, as defined by Desiraju in 1989 [38]. Molecular self-assembly is the driving force of crystal engineering, and supramolecular synthons defining crystalline packing can be either intermolecular interactions (e.g., hydrogen and halogen bonding) or coordination bonding. As such, crystal engineering comprises the study of polymorphs, hydrates/solvates, and different multicomponent crystal forms (salts, cocrystals, ionic cocrystals), according to the first approach (Figure 3). 

#### 2.1.1. Polymorphs

Polymorphs are defined as different crystalline arrangements of the same molecule in a crystal structure. The structural differences of two polymorphs can lead to different properties of a solid and, in the specific case of pharmaceuticals, can lead to differences in the pharmacological activity of the API.

In 2011, Duarte et al. published results obtained in a study of perindopril erbumine, an antihypertensive drug that comprises vasculoprotective and antithrombotic effects, in which two polymorphic forms were obtained by wet chemical methods, as well as a hydrate obtained both by solution and by LAG. The mechanochemical approach presented advantages in the preparation process, giving the same yield and purity as the solution method using only a few μL of water. The new hydrated form proved to be shelf-stable for several months and presented a similar dissolution profile as the commercial drug [39].

Gabapentin is a neuroleptic drug existing in three anhydrous polymorphic forms, as well as one hydrate and one chloride hemihydrate form. The thermal behavior and interconversion between the different polymorphic gabapentin forms were studied in solution and in the solid-state in a collaborative work of the Duarte and Braga research groups [40]. The solid-state techniques used were NG and LAG, and it was found that the transformation between polymorphic forms was accelerated in the presence of solvent.

#### 2.1.2. Screening and Synthesis of Multicomponent Crystal Forms

Mechanochemistry has further been demonstrated to be very efficient in the screening and synthesis of multicomponent forms since it expands simple methods not requiring the use of solvents [41]. Solid-state grinding allows for the formation of multicomponent forms using highly insoluble reagents that would be difficult to obtain with the traditional solution techniques [34,42,43]. Synthesis in the solid state provides a diversity of products, matching and even extending those observed in solution. In certain cases, mechanochemical synthesis delivers products that could not be obtained by wet methods, demonstrating how solid-state reaction facilitates supramolecular interactions. Importantly, the addition of catalytic amounts of a liquid to the grinding mixture further improves the efficiency of grinding [43].

**Figure 4:**
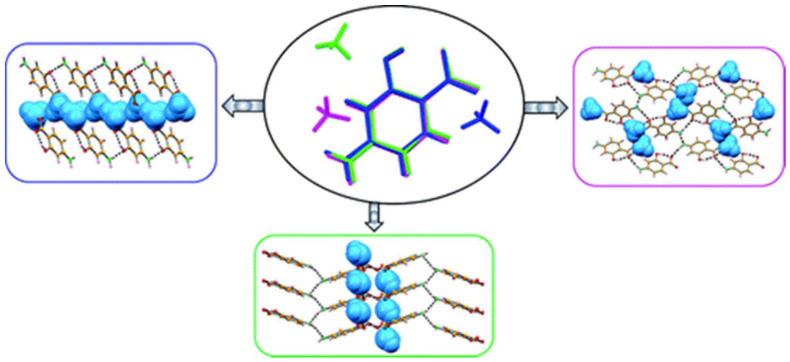
Ammonium 4-aminosalicylate salt polymorphic forms—Form I (left), Form II (right), and Form III (bottom), all displaying different supramolecular H-bond arrangements. Reproduction from ref. [46].

4-aminosalicylic acid (ASA) is an antibiotic used in the treatment of tuberculosis. André et al. [44] studied the reaction of ASA with six-membered ring coformers (dioxane, morpholine, and piperazine) to develop one solvate and three molecular salts as new acceptable pharmaceutical species of ASA. Different synthetic techniques were used in these studies, including both manual NG and LAG. The same group expanded the study on ASA cocrystals and salts by reporting new crystalline forms comprising diazabicyclo[2.2.2]octane (DABCO) and caffeine [45] by, again, recurring to different synthetic techniques. The new caffeine form was obtained exclusively by solution, as it is impossible to reproduce by mechanochemical methods. The reaction of ASA with ammonia yielded three polymorphic forms of the ammonium 4-aminosalicylate salt. When the reaction was conducted in solution, the three polymorphic forms were obtained concomitantly, while LAG and solid-gas reaction resulted in the formation of pure Form II of the 4-aminosalicylate salt (Figure 4) [46].

A screening of naproxen cocrystals exploring picolinamide, nicotinamide, isonicotinamide, and pyrazinamide as coformers was performed by R. Castro and colleagues, comparing the results obtained by the Kofler contact method and by LAG using a vibrational ball mill, aiming at a higher solubility of the final products. Naproxen is a nonsteroidal anti-inflammatory drug (NSAID) and belongs to Class II (high permeability, low solubility) of the Biopharmaceutics Classification System (BCS). The screening procedure led to the discovery of three new cocrystals: naproxen:picolinamide (1:1), naproxen:nicotinamide (2:1), and naproxen:isonicotinamide (1:1). Nevertheless, no experimental results were presented to confirm the solubility increase [47].

R. Castro was also involved in the study by Perpétuo et al. on the cocrystal screening of ketoprofen with nicotinamide by the Kofler contact method and by mechanochemistry using a mixer ball mill. Even though cocrystals were only obtained by the Kofler method, biological tests showed that the 1:1 ketoprofen-nicotinamide eutectic system obtained by grinding was more efficient as an anti-inflammatory than the pure ketoprofen [48].

The study of multicomponent forms of gabapentin, a neuroleptic drug, involved screening (solution and mechanochemistry methods, e.g., LAG) with several carboxylic acids [43]. In this study by André et al., mechanochemistry was again proven to be an important method, with terephthalic acid in particular, a highly insoluble starting material, yielding the new salt with a yield of approximately 100%, which is impossible to achieve by solution methods, even using very high-pH solutions.

Évora and colleagues [49] performed a cocrystal screening of the API lamotrigine, an antiepileptic drug, by NG using a vibration mill. The coformers used were theophylline, caffeine, picolinamide, nicotinamide, isonicotinamide, and diflunisal. The mechanochemical approach only generated physical mixtures of the starting materials; however, a thermodynamic approach using binary solid-liquid phase diagrams and theoretical simulations predicted the formation of cocrystals with theophylline, isonicotinamide, and a molecular salt with diflunisal.

Paracetamol is one of the most widely available analgesic and antipyretic APIs, and the problems arising from its poor tabletability performance are well known in the pharmaceutical industry [50]. André et al. [51] studied the cocrystallization of paracetamol with different coformers as a strategy to generate possible layered solid forms that would be thermodynamically stable and exhibit tablet-forming properties resembling those of polymorphic Form II; that is, the less stable polymorphic form of paracetamol presents the best tableting performance [50,52,53]. Solvent evaporation and manual LAG were used for cocrystal screening. Three solvates with morpholine were preferably obtained by solution techniques, while cocrystals with DABCO and cyclam were obtained by manual LAG [51]. All the new forms display very different supramolecular arrangements that will indeed influence their tabletability behavior, even though no experimental tests were performed.

Mid-infrared (MIR) spectroscopy, aided by chemometrics, was employed at line as a process analytical technology (PAT) tool for monitoring the synthesis of new lamivudine:theophylline cocrystals. Lamivudine is an HIV transcriptase inhibitor, and theophylline is a well-known member of the xanthine family present in coffee and tea and used as a drug for asthma treatment. NG and LAG with ethanol in a vibration mill led to different polymorphic forms of the cocrystal. The first led to polymorph II, and the second led to polymorph I, showing that the addition of solvent in very low quantities (μL) can influence the kinetics of the reaction, producing different results (cocrystal polymorphs). The concentration profiles retrieved by the chemometrics allowed for identification of the end of the reaction and understanding of the mechanism of formation of polymorphs I and II of the new cocrystal. It was found that polymorph I is synthesized by a rapid mechanism with a single intermediate, while the synthesis of polymorph II is performed through a slow mechanism with two intermediates [54].

Évora and colleagues described a structure-energetics study of binary crystal systems consisting of glycine (Gly), an amino acid, with fumaric acid (FA) or maleic acid (MA), two regioisomers with different proton-transfer abilities. Single-crystal X-ray diffraction experiments showed that the mechanochemically synthesized materials in which a vibration mill was used corresponded to a new FA:Gly (1:2) cocrystal and to a MA:Gly (1:1) salt that had been previously prepared by crystallization from solution [55].

Based on a thorough data mining, new multicomponent crystal forms of pipemidic acid, a first-generation quinolone antibiotic used to treat urinary tract infections, were explored with coformers enclosing carboxylic and sulfonic acid moieties. Successful results were obtained with glycolic, oxalic, and (R)- and (S)-camphorsulfonic acids [56]. Vibrational ball mills were used for successful synthesis by LAG. The new salts were obtained with high purity and yield.

André, in collaboration with Friščić´s team, disclosed novel cocrystals of ß-estradiol and estrone with 1,2-dimethylnaphthalene, phenanthrene, anthracene, 9,10-anthraquinone, phenanthridine, benzo[h]quinoline, and perfluoronaphthalene [57]. Knowledge of the molecular recognition and complexation of steroids was already an established area of research, and solid-state chemistry, along with crystal engineering, had recently been proposed to explore their molecular recognition properties[58]. The novel cocrystals obtained by LAG by Friščić and colleagues led to the conclusion that minor changes in the molecular structure of steroids may result in pronounced changes in its solid-state complexation, besides providing the first guidelines for developing new solid forms of ß-estradiol and estrone [57].

Évora et al. [59] applied mechanochemistry to prepare a multi-API cocrystal with pyrazinamide, a drug used to treat tuberculosis, and diflunisal, a nonsteroidal anti-inflammatory drug. Diflunisal is a low-solubility drug belonging to Class II of the BCS, and pyrazinamide has non-gouty polyarthralgia observed as a side effect, which is frequently treated with drugs such as diflunisal. The 1:1 pyrazinamide-diflunisal cocrystal was successfully prepared by different mechanochemical methods: annealing a mortar-ground mixture by thermal activation at 80 °C, room temperature annealing, neat ball-mill grinding, and ethanol-assisted ball milling (using a vibration mill). Ethanol-assisted grinding for 30 min at 15 Hz resulted in complete conversion to the cocrystal structure, while the neat process produced a low-crystalline material. Solution cocrystallization was also attempted and produced a mixture of the cocrystal with the pure APIs due to the different solubility between the two compounds, which gives rise to noncongruently saturating systems.

In 2018, Duarte and Grepioni reported [60] a study on a family of ionic cocrystals (ICC) of enantiopure L-proline and racemic DL-proline with LiX (X = Cl, Br, I), showing the chiral preference of Li^+^ for homochiral coordination with Cl and Br and racemates with Cl and I. Chiral resolution of racemic compounds is still an issue with enormous practical implications, particularly in the pharmaceutical industry. The attempts to gain control of the process that may lead from a racemic compound to separation of the enantiomers via crystallization of conglomerates might open new avenues. This paper provided evidence that coordination with Li^+^ cations of the amino-acid proline represents one such route.

#### 2.1.3. Improvement of Physicochemical Properties 

The improvement of physicochemical properties of APIs, e.g., solubility and stability, has been one of the main objectives for multicomponent synthesis.

Dapsone is an antibiotic used to treat several diseases, such as acne, rosacea, tuberculosis, and AIDS-related pneumonia. With the aim of improving its physicochemical properties, in particular, its solubility, solution and mechanochemical methods (LAG) were used to design new dapsone cocrystals by exploring N–H…O/N interactions using amide and pyridinic derivatives as potential coformers. Two new cocrystals with ξ-caprolactam (CAPRO) and 4,4′-bipyridine (BIP) were synthesized. In the case of CAPRO, the material obtained by solution and LAG were the same, but with BIP, the final product obtained by LAG was different from the one obtained by solution. Additionally, the authors found that the solubility of dapsone in the new forms was not improved when compared with the free crystalline drug [61].

**Figure 5:**
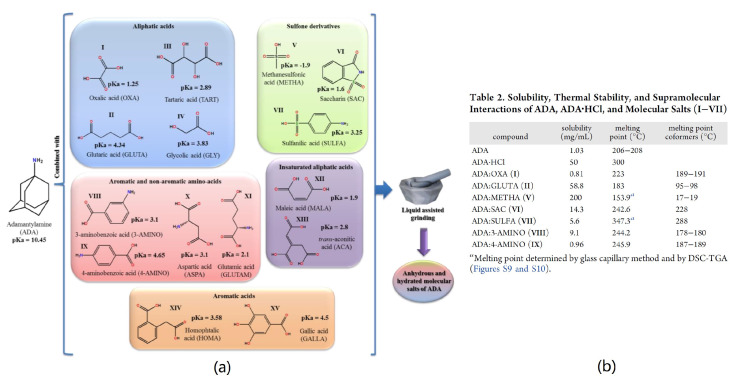
ADA new multicomponent crystal forms (**a**) and their melting point and solubility (**b**). Reproduction from ref. [62].

The reactivity of the neuroleptic drug adamantylamine (ADA) towards aliphatic carboxylic acids, sulfone derivatives, and aromatic amino acids was screened for the first time using simple mechanochemical methods: manual NG and LAG (Figure 5). Seven new molecular salt structures were reported. From those, six of them (the exception is the molecular salt with oxalic acid) presented higher solubilities than ADA, but only the molecular salts with glutaric and methanesulfonic acids are more soluble than the commercially available ADA·HCl [62].

**Figure 6:**
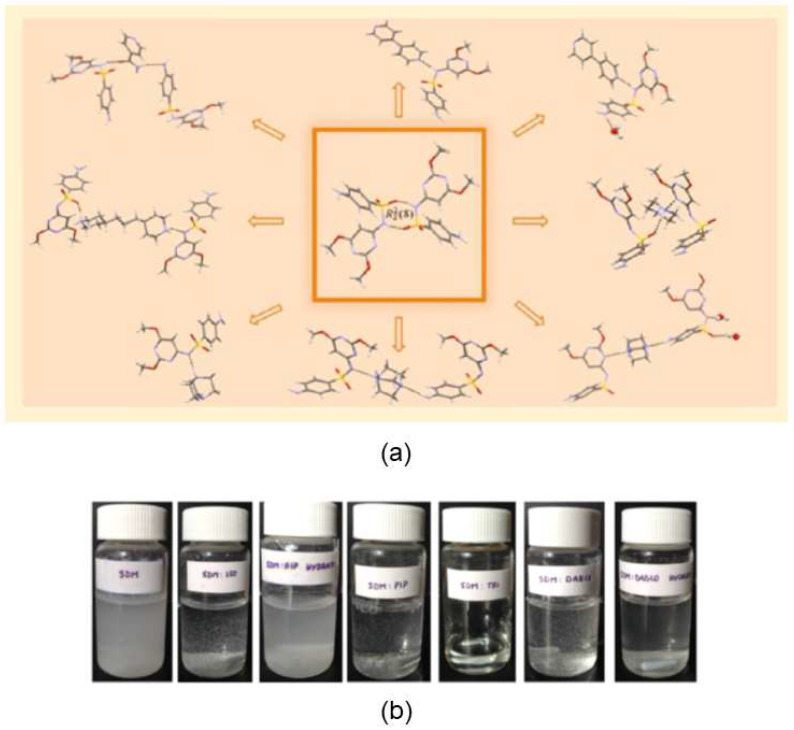
New sulfadimethoxine multicomponent crystal forms (**a**) and preliminary solubility studies (**b**). Reproduction from ref. [63].

The work of Domingos et al. [63] has shown that both wet chemical and mechanochemical synthetic procedures can lead to the same results. Eight new multicomponent forms were synthesized (two cocrystals and six salts) of sulfadimethoxine (SDM), an antibiotic commonly used in veterinary applications (Figure 6). The new forms were obtained both by solution crystallization and LAG, showing the preference for the formation of multicomponent crystal forms with amine derivatives. Cocrystals were unveiled with isonicotinamide (SDM:ISO) and 4,4′-bipyridine (SDM:BIP:Acetone; SDM:BIP:H_2_O), and molecular salts were synthesized with piperazine (SDM:PIP), 4,4′-trimethylenedipiperidine (SDM:TRI), and 1,4-diazabicyclo[2.2.2]octane (two anhydrous polymorphic forms (SDM:DABCO) and one hydrate (SDM:DABCO:H_2_O). Studies on the aqueous solubility of the resulting compounds revealed that molecular salts present higher solubility in water than cocrystals, an important aspect for the improvement of SDM performance.

**Figure 7:**
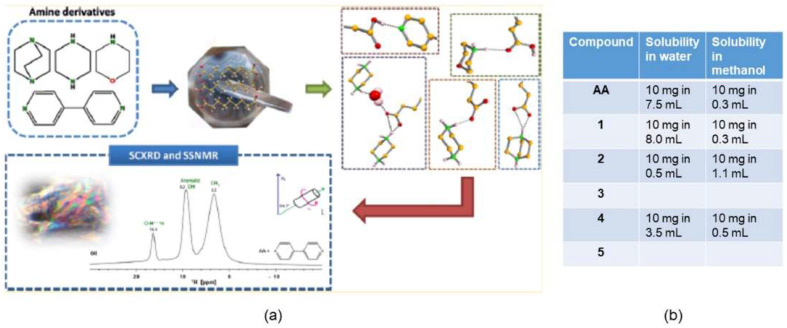
Azelaic acid new multicomponent crystal forms (**a**) and comparison of the solubility results in water and methanol (**b**). Reproduction from ref. [64].

New multicomponent forms of azelaic acid (AA), a compound that has antimicrobial and anti-inflammatory properties and is used to treat skin disorders, were synthesized with the objective of improving AA solubility. In this work, five new forms were obtained by LAG and conventional solution methods. The resulting crystalline forms (cocrystal with 4,4′-bipyridine) (AA:BIP); anhydrous and hydrated molecular salt with piperazine (PIP); and two anhydrous molecular salts with morpholine (MORPH) and 1,4-diazobicyclo[2.2.2]octane (DABCO) were independent of the synthetical procedure used [64]. The authors also studied the solubility of the new multicomponent forms and compared it with AA solubility, concluding that two of the molecular salts (AA:PIP (1:1); AA:MORPH (1:1)) were more soluble than AA and that the cocrystal with BIP (AA:BIP (1:1)) had a solubility similar to that of AA. The other two molecular salts were unstable, and therefore, their solubility was not determined (Figure 7).

**Figure 8:**
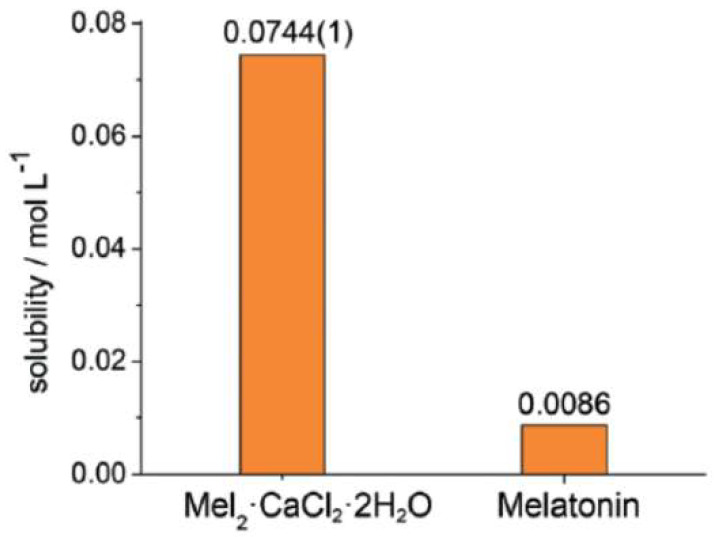
Comparison between the solubility in water at 293 K of melatonin and that of the ionic co-crystal mel_2_ CaCl_2_ 2H_2_O in mol L^−1^. Reproduction from ref. [65].

Two cocrystals of melatonin, a pineal hormone produced from the essential amino acid tryptophan mainly during the night, with piperazine and DABCO, as well as a hydrated ionic cocrystal with CaCl_2_, were obtained directly in the solid state by LAG using a few drops of ethanol in a vibration mill. The ionic cocrystal was also obtained by slurry in ethanol for two days. The thermodynamic solubility of the ionic cocrystal was determined, showing to be almost one order of magnitude higher with respect to pure melatonin, thus representing a potential new mode for oral melatonin administration (Figure 8) [65].

The formation of new salts with the natural antioxidant and geroprotector L-carnosine were explored by solution, vapor diffusion, and solid-state synthesis. A total of 11 new crystalline salts with several “generally regarded as safe” (GRAS) organic acids were obtained and characterized by X-ray powder and single-crystal diffraction, as well as thermal analysis. Additionally, an amorphous folate salt was obtained and characterized by Raman spectroscopy and thermal methods. Even though mechanochemistry encountered serious limitations in the synthesis of these forms, it was shown to be appropriate, at least for the system with fumaric acid. The folate salt may be of particular interest, and therefore, its solubility was tested, showing that it has similar solubility to that of the commercial sodium folate [66].

The encapsulation of montelukast (MLK), an oral antiasthmatic drug, with Υ-cyclodextrin (Υ-CD) by co-grinding using a mini planetary ball mill was performed to improve the stability of the API when exposed to water and light. The solid-state 1:1 inclusion compound, Υ-CD.MLK, was obtained by ball milling [67]. This work by Braga et al. showed how mechanochemistry can be used as a solvent-free methodology to form inclusion complexes in a short period. Using this technique, the drying step is avoided, improving the energetic input and preventing drug degradation through hydrolysis. Importantly, the authors claim that the complex preserves its stability on storage for at least 3 months.

In the work by Fernandes and colleagues, two new theophylline cocrystals with 4-aminosalicylic acid and 4-aminobenzoic acid were prepared by LAG with acetone in a ball mill to avoid the formation of the theophylline hydrate. Two ball mills were used: a vibration mill with two 10 mL vessels of stainless steel and a planetary ball mill with a 5 mL grinding bowl. Three stainless steel spheres of about 5 mm in width were added in the reactors on all the preparations [68]. No differences in product yield or crystallinity were found upon prolonged milling times (well beyond 1 h), nor by adding more solvent or using different mills.

#### 2.1.4. Non-Crystalline Multicomponent Forms

**Figure 9:**
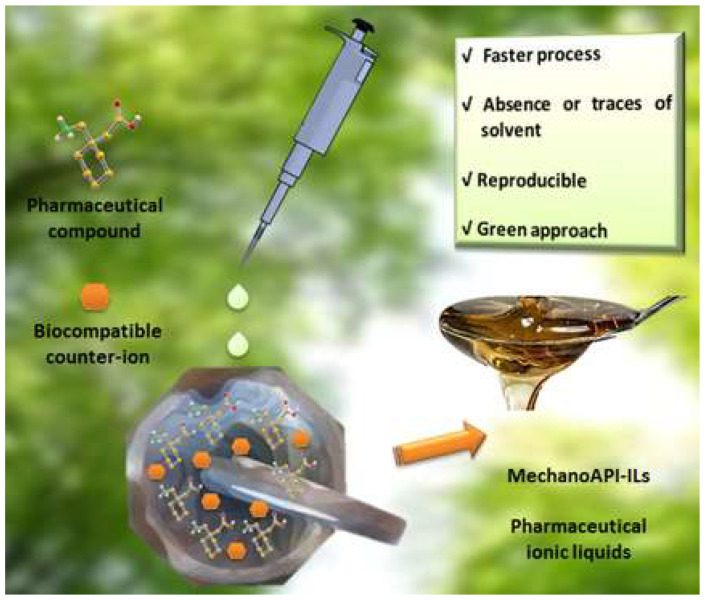
New pharmaceutical ionic liquids based on gabapentin. Reproduction from ref. [35].

Ionic liquids (ILs) are an emergent type of material with several advantages and are considered green solvents. Martins and colleagues [35] used mechanochemistry to synthesize six new API-ILs based on gabapentin and L-glutamic acid (Figure 9). The authors demonstrated that it is possible to synthesize ionic liquids with biological activity (API-ILs) through an eco-friendly solid-state technique. This mechanochemical approach overcomes some severe limitations of the conventional synthetic approach, such as the need to solubilize the API, long reaction times, and the presence of impurities.

Hot-melt extrusion (HME) was revealed to be a successful technology for a large spectrum of applications in the pharmaceutical industry. One of the most recently reported applications of HME is the stabilization of amorphous drugs through its incorporation into polymer blends in the form of amorphous solid dispersions (ASDs). Simões et al. [69] used HME to produce etravirine ASDs. The authors state that the results are promising and that the next steps will include formulation with additional excipients and the optimization of the HME process parameters. The same research group also used HME to prepare ASDs of ibrutinib [70]. Extrusion was also used to assess the co-extrudability of wet masses as a means of manufacturing a novel dosage form for oral administration of drugs with sustained releases over time of administration. Mono-, bi-, and tri-layer extrudates were manufactured and evaluated for extrudability, among other properties [71,72,73].

Although the majority of the work developed in Portugal has been intended for pharmaceutical applications, the synthesis of liquid crystals by mechanochemistry has also been explored. Liquid crystals are materials with interesting properties, with applications in televisions, computer monitors, and telephones, among others. For the supramolecular chemist, there is great interest in studying liquid crystal systems, especially those in which the combination of supramolecular interactions and molecular shape may provide a mesophase [74]. A series of thermotropic hydrogen-bonded liquid crystalline structures based on 4,4′-bipyridyl and aliphatic carboxylic acids was prepared by mechanochemistry using a ball mill [75]. The same research group reported the mechanochemical synthesis of four liquid crystals between 4-(octyloxy)benzoic acid and a 4-alkylbenzoic acids, obtained by mixing 1:1: molar ratios of 4-(octyloxy)benzoic acid and one of the various 4-alkylbenzoic acids in ball-mill system [74].

### 2.2. Mechanochemistry in the Synthesis of BioMOFs and Coordination Polymers

The advantages of mechanochemistry in the field of metallodrugs, metallopharmaceuticals, and bio-inspired metal-organic frameworks (BioMOFs) has also been unveiled by Portuguese research teams [76,77,78].

In 2009, Duarte and colleagues disclosed drug-containing coordination and hydrogen bonding networks of 4-aminosalicylic acid and piracetam by exploring manual LAG [33]. These systems showed that the coordination of active pharmaceutical ingredients to metals by mechanochemistry could indeed be an alternative approach in the search for new drugs and new delivery pathways.

Another important study was presented by André et al. in 2013, disclosing the mechanosynthesis of 10 new coordination networks of gabapentin with Y(III), Mn(II), and several lanthanides (LnCl_3_, Ln = La^3+^, Ce^3+^, Nd^3+^, Er^3+^) [79]. The structural diversity allowed for analysis of the different coordination modes of gabapentin to the metal sites: bidentate coordination (chelation), the “bridge” coordination; and “bidentate-bridge” coordination. Even though these compounds have been shown to be highly hygroscopic and unstable, this was another proof of concept supporting the application of mechanochemistry for the synthesis of new biomaterials.

Antibiotic Coordination Frameworks Obtained by Mechanochemistry

Within BioMOFs and metal complexes, antibiotics have been exploited as ligands seeking synergistic effects with different biocompatible metal sources [77]. This approach leads to antibiotic coordination frameworks (ACFs) and was motivated by the need to increase the efficiency of antibiotics that are becoming less effective due to antimicrobial resistance mechanisms.

Nalidixic and pipemidic acids are first-generation quinolone antibiotics mainly used to treat urinary tract infections and are effective against Gram-negative and Gram-positive bacteria, with nalidixic acid exhibiting higher efficiency against Gram-negative microorganisms. The coordination of these antibiotics to biocompatible metals emerged as an alternative approach to improve their biological and/or pharmaceutical activities while tuning their physicochemical properties.

**Figure 10:**
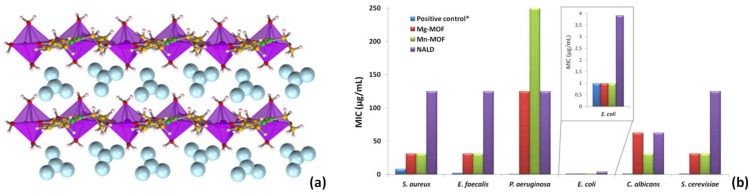
(**a**) Representation of the Zn(II)/Mg(II)-nalidixic acid 1D frameworks (metal centre represented in purple and polyhedral mode; nitrate counter ions represented in light blue and space filling mode—for the Mn(II) framework, nitrate is replaced by chloride); (**b**) Minimal inhibitory concentrations for the nalidixic acid Mn- and Mg-MOFs against Gram-positive (*Staphylococcus aureus* and *Enterococcus faecalis*) and Gram-negative (*Pseudomonas aeruginosa* and *Escherichia coli*) bacteria and yeasts (*Candida albicans* and *Saccharomyces cerevisiae*). (**b**) Reproduced from [77].

The coordination of nalidixic acid to Zn(II) [80], Mn(II), and Mg(II) [36], as well as its coordination to Cu(II) [81], was achieved by mechanochemical reactions. Structural elucidation revealed that it was possible to tune the LAG reaction conditions to obtain similar 1D MOFs with Zn(II), Mn(II), and Mg(II) that were stable and more soluble in water than nalidixic acid. Importantly, the examples with Mn(II) and Mg(II) have shown the great potential of this type of compound to increase the antimicrobial activity of commercially available antibiotics (Figure 10).

Exploring pipemidic acid as a ligand, mechanochemistry yielded four new complexes with Mn(II), Ca(II), Zn(II) and Cu(II), all of them characterized by the formation of extended hydrogen-bonded frameworks [56,82]. The Mn(II), Ca(II), and Zn(II) complexes are isostructural, and antimicrobial activity tests reveal that they are able to improve efficiency against *Staphylococcus aureus* and *Escherichia coli* without increasing the toxicity of pipemidic acid [82].

Azelaic acid is used to treat mild to moderate acne, and it is usually available as topical formulations, often affected by its low aqueous solubility. Quaresma et al. designed, synthesized, and characterized five new azelaic acid MOFs with Mg(II), K(I), and Na(I) [83]. It is worth noting that depending on the mechanochemical reaction conditions, different polymorphic forms were obtained with Mg(II) and with Na(I), with two different forms of each being reported. The new MOF structures lead to higher solubility, and antimicrobial activity tests show increased activity against *Staphylococcus aureus* and *Staphylococcus epidermidis*, two Gram-positive bacteria responsible for skin infections. The MOF based on potassium revealed a greater increase in solubility and has been shown to be the most active against both bacteria tested.

With pyrazinamide, an antibiotic used in the treatment of tuberculosis, NG led to the formation of four complexes with Zn(II), Mn(II), and Ag(I) [84]. By adjusting the stoichiometry of the starting materials, it was possible to obtain two different Ag(I) compounds. These novel complexes were tested against *E. coli*, *S. aureus*, and *M. smegmatis*, revealing promising results. The Ag(I) compounds that have shown to be particularly active against *M. smegmatis*, a non-pathogenic model organism of *M. tuberculosis*.

### 2.3. Mechanochemistry in/for Catalysis

**Figure 11:**
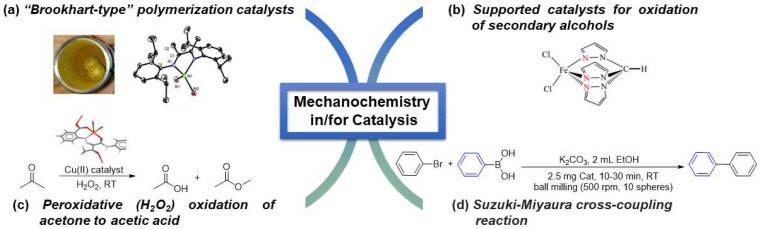
Applications of mechanochemistry in the synthesis of catalysts (**a**) and in catalytic reactions (**b**–**d**).

The application of mechanochemistry for the synthesis of catalysts has also been growing over the last years, mainly due to its ease of synthesis scalability, and sustainability, as well as the superior properties of the resulting materials [85]. In 2014, Gomes et al. [37] described the mechanochemical synthesis of the first Ni(II) and Co(II) complexes with the general formula [M(α−diimine)Br_2_] (M = Ni, Co). These Ni(II) complexes, which are generally known as Brookhart-type catalysts, were successfully prepared from the reaction of [NiBr_2_(DME)] (DME = 1,2-dimethoxyethane) with the appropriate α−diimine ligand, under NG conditions, in short reaction times (20 min), and with quantitative yields (Figure 11a). Moreover, the use of NiBr_2_ instead of [NiBr_2_(DME)] also led to the formation of the desired products in quantitative yields, although with slightly longer reaction times (30 min). Traditionally, the synthesis of these complexes is normally carried out in solution over 24 h, and when NiBr_2_ was employed, only a 67% yield could be obtained. When CoCl_2_ was used as the metal source, the preparation of the Co(II) derivatives was accomplished in only 20 min, maintaining the quantitative yields. The use of mechanosynthesis enabled not only a drastic reduction in the reaction time (from 24 h to 20 min) but also the possibility to easily obtain these complexes in quantitative yields, presenting mechanochemistry as a powerful synthetic technique and an alternative approach for the synthesis of coordination and organometallic compounds.

In 2017, Ribeiro et al. [86] reported the successful preparation of 3d metal (Cu, Fe, Co, V) containing composites with multiwalled carbon nanotubes and graphene oxide (CNTs and GO) additives. Their preparation was performed in a planetary ball mill using NG for 1 h at 450 rpm, with rotational inversions every 5 min. These composites acted as catalysts in the solvent-free, microwave-assisted oxidation of 1-phenylethanol to acetophenone, using tert-butyl hydroperoxide (TBHP) as oxidant, with yields up to 85% when a percentage (wt%) of CNTs of 5% was used. More recently, Soliman et al. [87,88] extended the application of mechanochemistry to the preparation of Pd(II) and Pt(II) composites with carbonaceous materials, namely activated carbon, multiwalled carbon nanotubes, and graphene oxide (AC, CNTs and GO), using NG conditions in a planetary ball mill. These derivatives, which showed metal-to-carbon ratios of 4.7 and 8.5 wt% for the AC composites, were employed as catalysts for the Suzuki-Miyaura cross-coupling reaction, using the model reaction between bromobenzene and phenylboronic acid under liquid-assisted grinding conditions (Figure 11d). The Pd/AC composites exhibited high mechanochemical catalytic activity, with molar yields up to 80% and turnover number (TON) and turnover frequency (TOF) of 222 and 444 h^−1^, respectively. Moreover, a yield of 26% (TON of 228 and TOF of 1.4 × 10^3^ h^−1^) was obtained after 10 min reaction for the AC-PT1 catalyst. This is an interesting result, as Pt(II) catalysts are not as common as Pd(II) catalysts for Suzuki-Miyaura reactions.

Matias and colleagues [89] developed the use of a magnetic core-shell support for a C-scorpionate metallic complex, and the final material was tested as catalyst for the oxidation of secondary alcohols using the model substrate 1-phenylethanol (Figure 11b). Interestingly, the peroxidative alcohol oxidation step using this material was tested with alternative energy sources: solution, conventional thermal heating, microwave-assisted oxidation, ultrasounds-assisted oxidation, and mechanochemical oxidation. The mechanochemical approach was carried out using a ball mill. Comparison of the results allowed for the conclusion that both sono- and mechanochemical oxidations lead to significantly lower amounts of oxidation product than the conventional thermal heating procedure. In 2020, Gurbanov and colleagues [90] reported, for the first time, the use of mechanochemistry for the synthesis of Cu(II)-arylhydrazone coordination polymers. Three novel Cu(II) coordination polymers resulted from recrystallization in different solvents of the bulk intermediate of the ball-milling reaction and were then isolated. The outcome of the mechanochemical synthetic route provided higher yields than the solution-based process. These compounds have shown to be effective homogeneous catalysts for the peroxidative oxidation of acetone to acetic acid and methyl acetate, with yields of up to 50% at room temperature (Figure 11c). Thus, mechanochemistry has contributed to the extension of the application of arylhydrazone complexes as catalysts.

### 2.4. Mechanochemistry in the Synthesis of Organic and Inorganic Compounds

**Figure 12:**
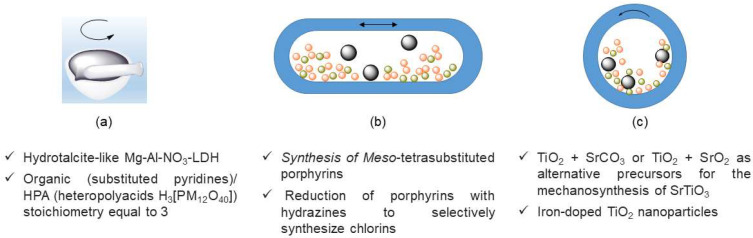
Mechanosynthesis of organic and inorganic compounds using manual grinding (**a**), planetary ball milling (**b**), and vibration ball milling (**c**).

Mechanochemistry has been widely employed in the synthesis of inorganic and organic compounds, proving to be a powerful, low-cost, and green method with significant benefits in relation to conventional synthetic methodologies. In the early stages of mechanochemistry in Portugal, L. Mafra collaborated with A. N. Ay and B. Zümreoglu-Karan in the solid-state NMR characterization of layered double hydroxides (LDH) [91]. These hydrotalcite-like Mg-Al-NO_3_-LDHs were prepared by manual grinding of hydrated magnesium and aluminum nitrate salts with sodium hydroxide (Figure 12a) in short reaction times (5–10 min). LDH samples were also prepared from a solution-based method by co-precipitation, leading to products with similar characteristics as those obtained by mechanochemistry but displaying lower Mg/Al ratios.

In 2012, Monteiro and colleagues [92] developed work on the thermodynamic restrictions observed in the mechanosynthesis of strontium titanate. The authors used TiO_2_ + SrCO_3_ or TiO_2_ + SrO_2_ as alternative precursors for the mechanosynthesis of SrTiO_3_, using anatase combined with the corresponding stoichiometric quantities of strontium carbonate or strontium peroxide under NG conditions in a planetary ball mill (Figure 12c). This work showed that this reaction is only feasible under highly energetic milling. The use of mixtures of anatase + SrO_2_ reactants that ensure sufficient thermodynamic conditions led to more efficient and faster mechanosynthesis.

In the field of inorganic materials, in particular, nanoparticles, Carneiro et al. [93] reported the synthesis of iron-doped TiO_2_ nanoparticles using a process based on ball milling (Figure 12c) and photocatalytic properties of the newly synthesized doped-nanoparticles. The authors concluded that for milled TiO_2,_ the correlation between the process parameters and the structural, morphologic, optical, magnetic nanoparticles showed that: (a) the TiO_2_ phase composition is strongly dependent on the ball-milling rotation speed; (b) the ferromagnetic behavior and the saturation magnetization values decrease with the increase in the ball-milling rotation speed; and (c) the absorption of the dyes may be enhanced due to their greater surface area.

In 2017, a bridge between inorganic and organic synthesis was established by Santos and colleagues [94]. The authors synthesized inorganic/organic hybrid compounds from the reaction between hydrated heteropolyacids (HPA) H_3_[PM_12_O_40_]. *n*H_2_O (M = Mo, W) and substituted pyridines with different sizes and functionalities, using manual NG for 20 min, led to derivatives with organic/HPA stoichiometry equal to 3 (Figure 12a). Moreover, solution-based synthesis was also performed for comparison purposes, showing that the compounds obtained by mechanochemical synthesis displayed enhanced charge transfer and nonlinear optical properties.

In recent years, some examples of mechanochemical synthesis of porphyrin derivatives were reported by Pineiro and colleagues [95,96] (Figure 12b). *Meso*-tetrasubstituted porphyrins were synthesized by one-step and two-step approaches, i.e., by separating or not separating the cyclization of pyrrole and aldehyde to form porphyrinogen from its oxidation to porphyrin, using mechanochemistry in a vibration ball mill [96]. Several reaction conditions were tested, the best results being obtained for the two-step procedure using liquid-assisted grinding in the oxidation step and MnO_2_ as a heterogeneous oxidant. Sustainability was also assessed by the E-factor and EcoScale, two sustainability metrics, which allowed for a comparison between the two used methods. In the search for more sustainable processes for the reduction of *meso*-tetraarylporphyrins, the authors decided to test the mechanochemical reduction of porphyrins with hydrazines to selectively synthesize chlorins [95]. The procedure consisted of the hydrogenation of porphyrins using diimide generated and consumed under mechanical activation, under neat grinding conditions. The use of hydrazine hydrate as diimide source led to high conversion, high selectivity, and nearly ideal sustainability scores (atom economy of 97% and E-factor of 0.96).

In 2019, Emmerling and colleagues, including Minas da Piedade, were involved in mechanistic studies of the mechanosynthesis of ettringite [97]. Ettringite ([Ca_3_Al(OH)_6_]_2_·(SO_4_)_3_·26H_2_O) is an important mineral present in cement concretes, with technological potential due to its ion exchange ability. Its synthesis by mechanochemical methods had already been reported [98], and in this second work, the authors focused their attention in determining the mechanistic effects involved in this synthetic approach. Mechanochemistry reveals high conversion and short reaction times (94% in 2 h), without CO_2_ release or wastewater production nor the need for product separation, contrary to the conventional wet-chemical traditional synthesis. Mechanistic studies were performed based on one-, two-, and three-step synthetic procedures, and the results provided a new understanding of mechanochemical ettringite formation, important for further improvement of synthesis efficiency, envisaging a possible industrial implementation [97].

In 2020, Pineiro and colleagues [99] reported an automatic, homemade, single-screw device consisting of an electric motor, a drill, and a drill chamber for applications in mechanochemistry. The development of the device arose from the fact that the available ball-milling systems required the control of several variables that could prevent reproducibility of the experiments. The new single-screw device showed a high applicability and versatility in the synthesis, with high yields at room temperature and short reaction times of derivatives such as chalcones, dihydropyrimidinones, dihydropyrimidinethiones, pyrazoline, and porphyrins.

## 3. Final Remarks

The history of mechanochemistry in Portugal is still recent, dating back to 2009. However, a lot of significative work has already been disclosed in a wide range of fields. Examples using NG, LAG, and ILAG via manual grinding, vibrational ball milling, and hot-melt extrusion have been reported.

The development of new crystal forms of pharmaceutical compounds based in crystal engineering and supramolecular chemistry is undoubtedly the area where more work has been carried out. Several studies show the possibility of using mechanochemistry for the synthesis of polymorphs, solvates, salts, cocrystals, and ionic cocrystals towards improved physicochemical properties. BioMOFs and ACFs prepared by mechanochemistry in quantitative yield and short reaction times have been reported as alternative drug forms with enhanced solubility and/or efficiency.

Nevertheless, other non-pharmaceutical-related areas have also been explored, with significant results. Topics such as ionic liquids and liquid crystals have also benefited from the application of this synthetic procedure. The preparation of catalysts by mechanosynthesis was accomplished in very short reaction times and in quantitative yields, and their application in catalytic reactions showed effective results both in homo- and heterogeneous media. Moreover, syntheses of a variety of organic and inorganic derivatives were also performed by several research groups, proving mechanochemistry as a powerful and green methodology with significant benefits in relation to conventional solution-based methods.

## Data Availability

Not applicable.

## Abbreviations

AA	Azeleic Acid
AC	Activated Carbon
ACFs	Antibiotic Coordination Frameworks
ADA	Adamantylamine
AIDS	Acquired Immunodeficiency Syndrome
API	Active Pharmaceutical Ingredient
ASA	4-Aminosalicylic Acid
ASD	Amorphous Solid Dispersion
BCS	Biopharmaceutical Classification System
BioMOFs	Bio-inspired Metal-organic Frameworks
BIP	4,4′-Bipyridine
CAPRO	ξ-Caprolactam
CNTs	Carbon Nanotubes
COFs	Covalent-Organic Frameworks
CPs	Coordination Polymers
DABCO	Diazabicyclo[2.2.2]octane
DME	1,2-Dimethoxyethane
FA	Fumaric Acid
Gly	Glycine
GO	Graphene Oxide
GRAS	Generally Regarded as Safe
HIV	Human Immunodeficiency Virus
HME	Hot-melt Extrusion
HPA	Heteropolyacids
ICC	Ionic Cocrystals
ILAG	Ion- and Liquid-assisted Grinding
ILs	Ionic Liquids
ISO	Isonicotinamide
IUPAC	International Union of Pure and Applied Chemistry
LAG	Liquid-Assisted Grinding
LDH	Layered Double Hydroxides
MA	Maleic Acid
MIR	Mid Infrared Spectroscopy
MLK	Montelukast
MOFs	Metal-Organic Frameworks
MORPH	Morpholine
NG	Neat Grinding
NMR	Nuclear Magnetic Ressonance
NSAID	Nonsteroidal Anti-Inflamatory Drug
PAT	Process Analytical Technology
PIP	Piperazine
PMMA	poly(methyl)methacrylate
POLAG	Polymer-Assited Grinding
SDM	Sulfadimethoxine
TBHP	Tert-butyl Hydroperoxide
Teflon	Polytetrafluoroethylene
TOF	Turnover Frequency
TON	Turnover Number
TRI	4,4′-trimethylenedipiperidine
TSE	Twin Screw Extrusion
Υ-CD	Υ-cyclodextrin
